# Reference percentiles for children and adolescents for the digital motor performance test (DigiMot): results from the COMO-study

**DOI:** 10.1038/s41598-026-40270-7

**Published:** 2026-02-16

**Authors:** Thorsten Klein, Annette Worth, Claudia Niessner, Samuel Merk, Anke Hanssen-Doose

**Affiliations:** 1 Institute of Movement and Sport, Karlsruhe University of Education, Bismarckstraße 10, 76133 Karlsruhe, Germany; 2 Institute of Sports and Sports Science, Karlsruhe Institute of Technology, Kaiserstraße 12, 76131 Karlsruhe, Germany; 3 Department of Empirical Educational Research, Karlsruhe University of Education, Bismarckstraße 10, 76133 Karlsruhe, Germany

**Keywords:** Health care, Medical research

## Abstract

Responding to the growing demand for scalable assessment tools, this study provides the first age- and sex-specific reference percentiles for remotely assessed physical fitness tests in German children and adolescents aged 7-15 years. Data from 1149 participants (554 male; 595 female) of the COMO-study were used to assess coordination, muscular endurance, and flexibility via videoconference with the DigiMot test profile. Percentile curves were generated using generalized additive models for location, scale, and shape. Results revealed typical developmental trajectories, with performance increasing with age and differing by sex and fitness component. An exception was the decline in girls’ Push-up performance with age. Boys outperformed girls in Push-ups and Sit-ups from early age, while girls initially surpassed boys in coordination until early adolescence. Girls (mean: 77.5%) achieved better results in the Stand-and-Reach across all age groups than boys (mean: 48.1%). Performance variability was generally higher in boys across all tests. These findings provide a foundation for age- and sex-specific reference values for remotely assessed physical fitness. By enabling standardized interpretation of remote test results, the percentiles support broader applications of remote fitness diagnostics in research and practice despite some limitations in sample representativeness and percentile stability in older age groups.

## Introduction

Physical fitness is a key indicator of physical, cognitive, and neurological development in children and adolescents. Its early and continuous assessment is crucial not only for tracking individual growth but also for identifying early signs of developmental or health-related concerns^1–3^. Physical fitness in youth is associated with a wide range of health outcomes^4^, including reduced risk of obesity^5^, cardiovascular diseases^6^, and mental health^7^, as well as enhanced cognitive functioning and academic achievement^8^. Given these associations, continuous monitoring of physical fitness is essential to detect negative trends early and enable timely public health interventions, especially in light of global evidence showing declines in youth fitness levels^9^.

Physical fitness has traditionally been assessed through laboratory-based and field-based methods. Laboratory tests provide highly accurate and objective data using specialized equipment, while field assessments are more suitable for large-scale applications and are typically structured into standardized test batteries or profiles targeting various fitness components. However, both approaches present challenges in terms of scalability, accessibility, and logistical effort, which can limit their feasibility for population-wide monitoring. In recent years, advancements in digital technology have enabled the emergence of remote fitness assessments^10^, offering new opportunities for scalable, accessible, and efficient monitoring. As digital tools become increasingly integrated into health^11^ and educational^12^ settings, there is a growing demand for solutions that can evaluate physical fitness remotely in a standardized and child-friendly manner. The Digital Motor Performance Test (DigiMot)^10^ addresses this need by providing a structured remote assessment through short fitness tasks that capture key components of both health-related (muscular endurance) and skill-related (coordination) physical fitness^13^, while reducing the logistical burden associated with traditional field-based testing. Thus, DigiMot expands the range of feasible testing options, particularly in contexts where in-person testing is impractical, such as during large-scale online surveys or periods of restricted access like the COVID-19 pandemic.

To effectively track physical fitness trends over time, normative data are needed for the specific test items used. These reference values are essential to interpret individual fitness results, benchmark them against the general population, and support early identification of developmental delays. Widely used field-based test batteries such as Eurofit^14^, ALPHA^15^, YFIT^16^, and the MoMo test profile^17^ have played a central role in establishing such reference systems. Studies such as Tomkinson et al. (2018)^18^ and Ortega et al. (2023)^19^ provided comprehensive percentile values across Europe, enabling cross-country comparisons and large-scale health surveillance. Most notably, Niessner et al. (2020)^20^ presented representative, age- and sex-specific percentile curves for physical fitness across early childhood to early adulthood in Germany. Building on this foundation, the present study aims to extend the percentile-based monitoring approach to the digital domain by developing age- and sex-specific reference percentiles for the remote physical fitness test DigiMot in children and adolescents aged 7 to 17 years using data from the COMO-Study.

## Methods

### Study and sample

**Fig. 1:**
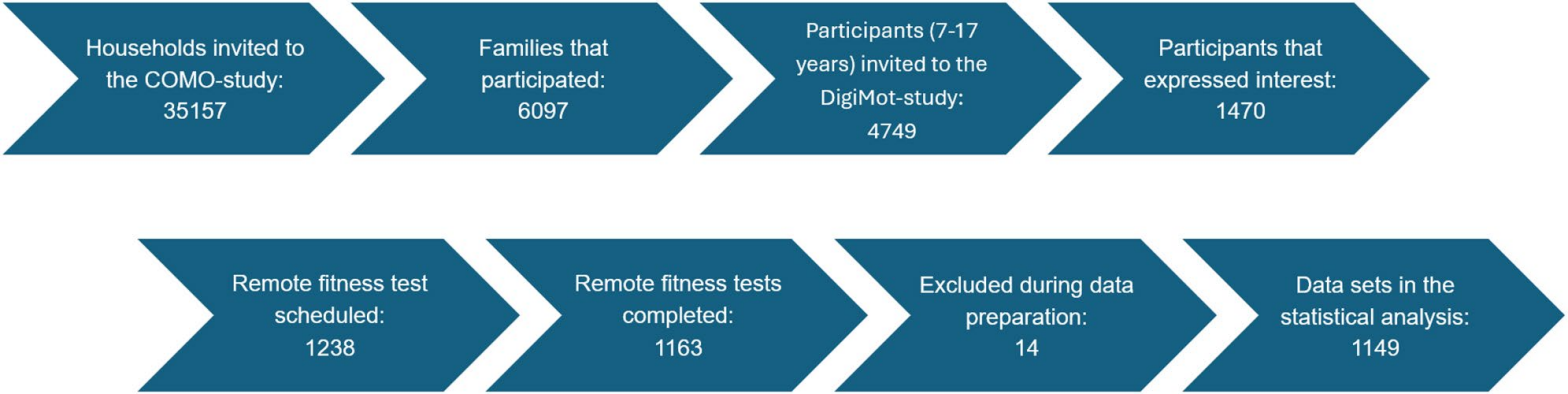
Participant selection flow chart of the DigiMot-study.

The COMO-study is a multicenter, interdisciplinary panel study with a prospective longitudinal design, collecting data in three annual waves (fall 2023, 2024, and 2025)^21^. It investigates changes in physical and mental health as well as health behaviors among children and adolescents aged 4 to 17 years in Germany in the aftermath of the COVID-19 pandemic, while accounting for socioecological contexts^21^. The first wave (COMO-1) took place between October 2023 and February 2024 and included children and adolescents living in private households across Germany as its target population. Sampling followed a two-stage, register-based procedure: municipalities were first selected proportionally by federal state, followed by a random draw of household addresses from local population registries^21^. In total, 35157 households were invited to participate, resulting in 6097 families completing at least one of the study questionnaires^21^. The DigiMot substudy was embedded within COMO-1 and focused on assessing physical fitness in a remote setting (videoconference). Participants for the DigiMot substudy were recruited through the COMO-1 questionnaires (Figure 1).

Only children and adolescents aged 7 to 17 years were eligible to take part in the DigiMot substudy. All participants interested in the substudy were first contacted by the study director and subsequently by the test administrators, which were trained prior to data collection and received standardized instruction in the test administration, to schedule an individual appointment for the remote fitness test. Access details for the Zoom meeting and the test mat were then sent to the participants, and the remote physical fitness test was conducted on the agreed date. In total, 1164 remote physical fitness test were completed. After data preparation (e.g. matching participants characteristics with the COMO-study) for the statistical analysis, data of 1149 children and adolescents aged 7 to 17 years remained. All relevant sample characteristics of these are presented in Table 1. More girls (51.8%) than boys (48.2%) participated in the study and the mean age of participants was 11.4 years. Regarding the individual age groups the participation rates between the ages 7 and 12 years were very high, but then dropped increasingly.

**Table 1:** Study sample characteristics.

Variables		Study sample
Sample size [N]		1149
Age (mean ± SD) [Years]		11.4 (2.8)
Sex (male/female) [%]		48.2 / 51.8
BMI (mean ± SD) [$kg/m^{2}$]		17.88 (3.26)
Jumping sideways [N]		1146
Push-ups [N]		1140
Sit-ups [N]		1137
Stand-and-reach [N]		1147
Age groups (male/female) [N]		62/63
7 years		62/63
8 years		80/80
9 years		65/94
10 years		69/61
11 years		66/60
12 years		62/61
13 years		45/49
14 years		33/39
15 years		31/36
16 years		26/39
17 years		15/13

### Remote physical fitness assessments

To remotely assess the physical fitness of participants in the DigiMot substudy the digital motor performance test DigiMot^10^, which is based on the MoMo test profile^17^ and was first developed during the COVID-19 pandemic, was used. For adaptation to videoconference-based administration, test selection emphasized feasibility and safety in a home environment, considering space and technical requirements. The DigiMot test contains four physical fitness tests assessing coordination, muscular endurance, and flexibility: (1) Jumping sideways (number of side-jumps between two fields in 15 seconds; average of two valid trials), (2) Push-ups (number of push-ups in 40 seconds; one trial), (3) Sit-ups (number of sit-ups in 40 seconds; one trial), and (4) Stand-and-reach (reached ground level or not; two trials).

All four fitness tasks have been validated in face-to-face assessments as part of the MoMo test profile^22^ and in remote videoconference-based conditions as part of the COMO-study^23^. Participants received a non-slip, foldable test mat (183 $\times$ 61 $\times$ 0.4 cm; Yogistar, Wiggensbach, Germany) to standardize conditions. Assessments were conducted via Zoom, allowing synchronized timing and optional video recording for quality assurance. In total, 824 of the 1163 completed remote fitness test were recorded (70.85%). The recordings of uncertain performances were re-evaluated by a second test administrator to verify results. This affected a total of 69 tests, of which 49 could be re-evaluated. The interrater reliability of the overall quality assurance process has been examined previously and demonstrated good to excellent agreement^23^. At the beginning of each session, parental consent for recording was obtained, and participants provided basic anthropometric (body height and weight) and health information. Before the fitness test was conducted, participants prepared the test setup in collaboration with the test administrator. Whenever feasible, the camera was positioned to capture the full body and the entire test mat. If this was not possible due to lack of space, the camera was adjusted after consideration by the test administrator to ensure maximal body visibility and continuous visibility of the test mat. Further details on test setup and implementation are available in the DigiMot test manual^10^. Each fitness task was explained using standardized instructional videos and demonstrated live by the test administrator. Participants completed practice trials, except for the Stand-and-Reach task, to ensure correct execution.

### Statistical analyses

The statistical analysis approach was adapted from Ortega et al. (2023)^19^ and Niessner et al. (2020)^20^ for all physical fitness tests except the Stand-and-reach test since performance was measured dichotomous (ground level reached or not) and therefore classical percentiles are not equally informative. The Stand-and-reach performance distributions were descriptively analyzed regarding sex and age groups. To prepare the data of the other fitness tests for analysis, first implausible values (e.g. zero values) were removed. Then extreme outliers were identified and excluded. Specifically, for each fitness test, a multivariate regression model was specified with the fitness test as the dependent variable. Age (modeled as a cubic spline with 5 degrees of freedom), sex, and their interactions were included as predictors. From this model, studentised residuals were calculated, and observations with values exceeding three standard deviations from the mean were removed. Further outliers were removed during the analyses based on the Q-statistics. To generate percentile curves and corresponding reference values, we employed generalised additive models for location, scale, and shape (GAMLSS)^24^. Multiple continuous distribution families, including the Box-Cox Cole and Green (BCCG), Box-Cox Power Exponential (BCPE), Box-Cox-t (BCT), and the generalised inverse Gaussian (GIG) distribution, were evaluated. The BCCG distribution corresponds to the traditional LMS method^25^, while the BCPE and BCT models extend this by incorporating an additional parameter $\nu$ (nu) to capture kurtosis ($\nu$ = 2 BCPE and BCCG coincide). Power transformations of age using values of $\lambda$ = 1/3 and $\lambda$ = 1/2 were also applied during model fitting. To enhance the representativeness of the subsample used for modeling (children aged 7-17 years), we applied the total survey weights provided in the main COMO study dataset. These weights incorporated both design weights and adjustment weights (including trimming of the top and bottom 2.5% of the original distribution to reduce sampling variances^26^), and were originally constructed for the full sample of participants aged 4 to 17 years. Therefore, we re-normalized the original weights so that their sum equaled the size of the analytic sample to preserve the relative weight structure and ensure appropriate scaling for use in the GAMLSS modeling. Model parameters were estimated using P-splines, and the optimal degrees of freedom were selected based on the Schwarz Bayesian Criterion (SBC^27^). Analyses were conducted separately for males and females, with the best-fitting model for each sex and fitness test determined using SBC. We concluded that the fit is generally reasonable by inspecting worm plots^28^, examining the Q-statistics^29^, and comparing predicted with empirical centiles. Only the resulting percentiles for the ages 7 to 15 years will be reported since the participation rate in the older age groups is very low and the results are therefore not reliable (Table 2). All analyses were run using functions of the the GAMLSS package^30^ in R (Version 4.5.1)^31^.

**Table 2:** GAMLSS model distributions, Schwarz Bayesian Criterions (SBC), removed outliers, sample sizes (N), and degrees of freedom (rounded to the third decimal place) for the parameters lambda ($\lambda$), mu ($\mu$), sigma ($\sigma$), and nu ($\nu$) for each physical fitness test by sex.

Physical fitness test	Sex	Implausible values removed	Outliers removed (Residuals/Q-statistics)	Distribution	N	$\lambda$	$\mu$	$\sigma$	$\nu$	SBC
Jumping sideways	Boys	2	2 (2/0)	BCCG	550	1/2	2.592	2.002	2.000	3776.434
Jumping sideways	Girls	1	15 (2/13)	BCCG	579	1/3	2.928	2.002	2.000	3565.824
Push-ups	Boys	5	0 (0/0)	BCCG	549	1/3	2.000	2.002	2.000	2975.190
Push-ups	Girls	4	7 (4/3)	BCCG	584	1/2	2.000	2.002	2.000	2814.686
Sit-ups	Boys	6	1 (1/0)	BCCG	547	1/3	3.263	2.002	2.000	3533.540
Sit-ups	Girls	6	3 (3/0)	BCCG	586	1/2	2.729	2.002	2.254	3280.191

## Results

**Fig. 2:**
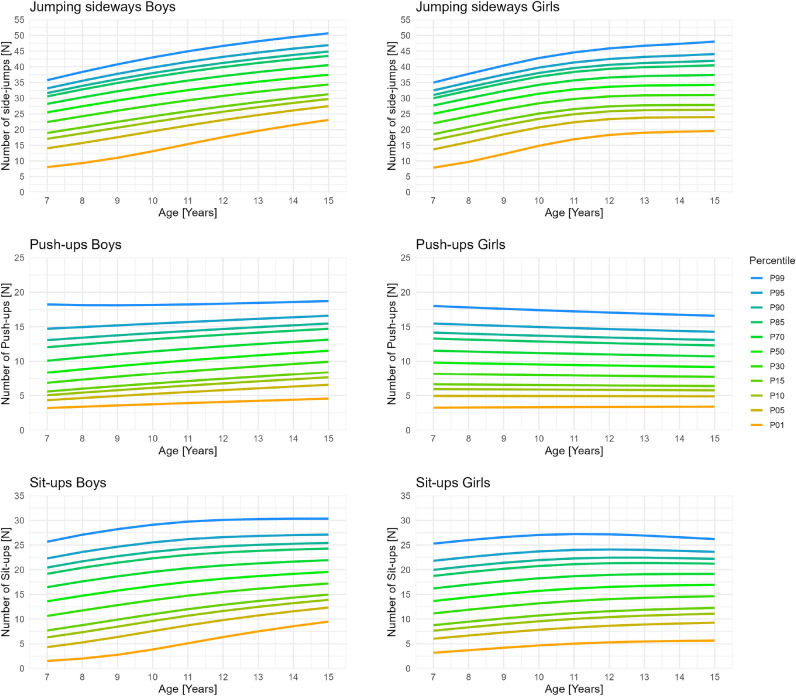
Reference percentile curves for coordination (Jumping sideways) and muscular endurance (Push-ups and Sit-ups) among German boys and girls aged 7 to 15 years.

**Fig. 3:**
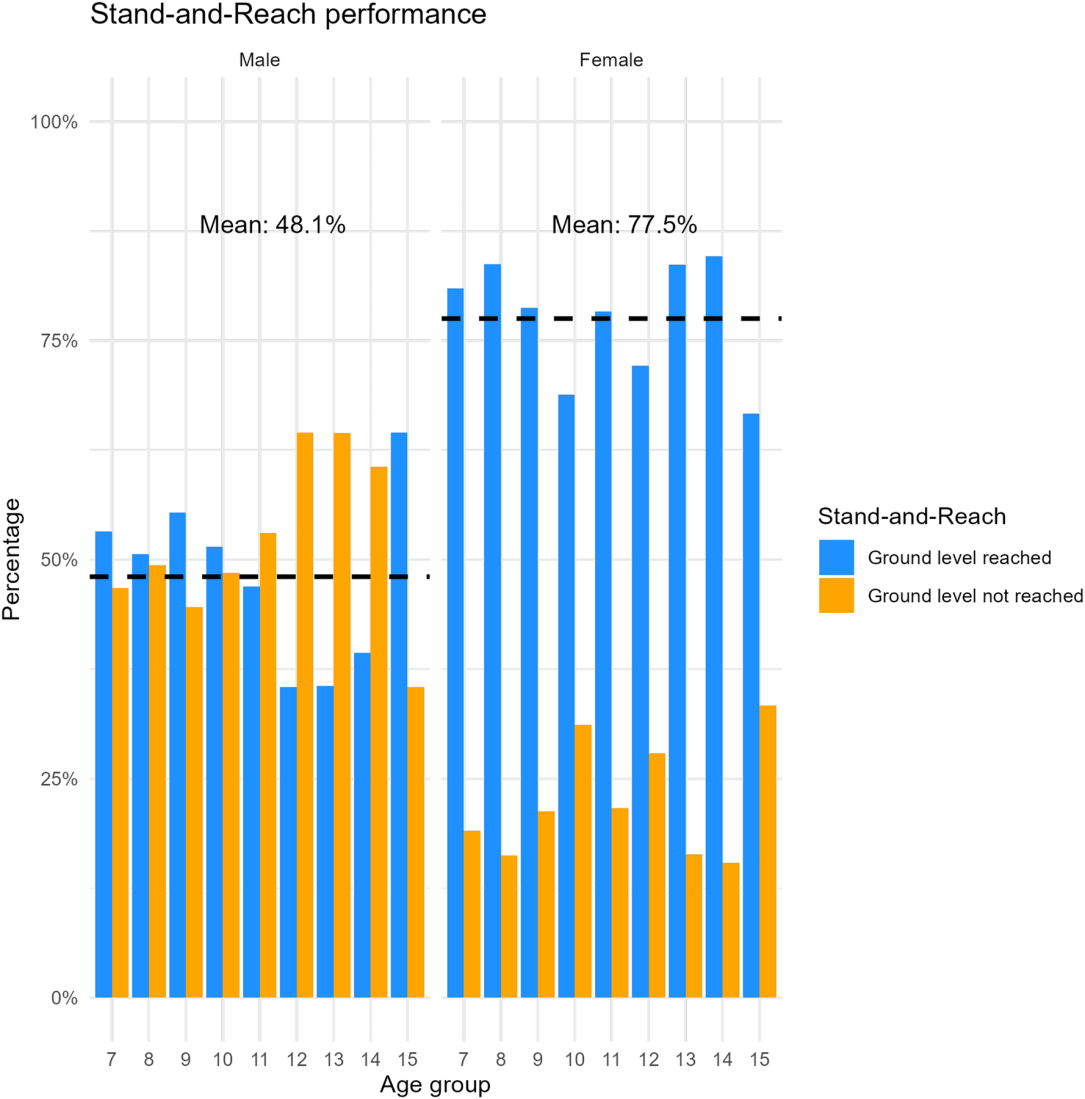
Stand-and-Reach performances in boys and girls aged 7 to 15 years.

After removing implausible values and outliers 1129 results for the Jumping sideways and 1133 results for the Push-ups and Sit-ups remained. Using these data, reference values and percentile curves were calculated for boys and girls aged 7 to 15 years. The best fitting models with the amount of removed implausible values and outliers, respective distributions, SBC, sample sizes, and the degrees of freedom for all parameters are presented in Table 2. Percentile values (Table 3) and curves (Figure 2) for the Jumping sideways show that both girls and boys increase their performance with age. In comparison girls on average perform equally or slightly better till the age of 12 years, after which boys perform better, since the increase in performance decreases more sharply for girls than for boys. Additionally, the average variation between the least fit (e.g., percentiles 1–10) and the fittest (e.g., percentiles 90–99) is on average across the age groups larger for boys compared with girl. Percentile values (Table 4) and curves (Figure 2) for the Push-ups show that performance development in boys and girls differ greatly. Girls performance decreased steadily with age, while boys performance increased steadily with age. Boys on average perform better in the Push-ups then girls at around the age of 10 years. Furthermore, the average variation between the fittest (e.g., percentiles 90–99) is on average across the age groups larger for boys while the average variation between the least fit (e.g., percentiles 1–10) is equal between boys and girls. Percentile values (Table 5) and curves (Figure 2) for the Sit-ups show that performance in boys increases steadily with age while girls performances begin to stagnate and even decline around the age of 10 years. On average boys perform better in the Sit-ups then girls at around the age of 8 years. Moreover, the average variation between the least fit (e.g., percentiles 1–10) and the fittest (e.g., percentiles 90–99) across the age groups is larger for boys. The performance distributions for the Stand-and-Reach test (Figure 3) show that girls outperform boys in all age groups, with an average of 77.5% of girls reaching ground level compared to 48.1% of boys. Boys’ performance declines at age 12 but improves again by age 15.

**Table 3:** Reference percentiles (P01 refers to the 1st percentile; other percentiles are denoted similarly) for coordination remotely assessed by the Jumping sideways test (number of repetitions in 15s) for children and adolescents from the COMO-Study (Boys: N=550; Girls: N=579).

	P01	P05	P10	P15	P30	P50	P70	P85	P90	P95	P99
*Boys ages [years]*											
7.00	8.08	14.06	17.08	18.94	22.42	25.50	28.21	30.58	31.64	33.14	35.75
7.50	8.67	14.89	17.95	19.83	23.35	26.49	29.27	31.72	32.82	34.37	37.09
8.00	9.35	15.75	18.83	20.72	24.27	27.45	30.29	32.81	33.94	35.55	38.38
8.50	10.14	16.66	19.72	21.61	25.17	28.38	31.27	33.85	35.02	36.69	39.61
9.00	11.04	17.59	20.62	22.50	26.05	29.29	32.22	34.86	36.06	37.77	40.79
9.50	12.04	18.54	21.52	23.38	26.91	30.16	33.13	35.82	37.05	38.80	41.92
10.00	13.13	19.48	22.41	24.24	27.74	31.00	34.01	36.74	37.99	39.79	42.99
10.50	14.26	20.42	23.28	25.07	28.55	31.81	34.84	37.61	38.89	40.72	44.00
11.00	15.40	21.34	24.12	25.88	29.32	32.58	35.62	38.43	39.72	41.59	44.94
11.50	16.52	22.22	24.93	26.65	30.05	33.30	36.36	39.20	40.51	42.41	45.83
12.00	17.61	23.07	25.70	27.39	30.75	33.99	37.06	39.92	41.24	43.17	46.65
12.50	18.65	23.88	26.45	28.11	31.42	34.64	37.71	40.59	41.93	43.88	47.42
13.00	19.63	24.66	27.16	28.79	32.06	35.26	38.33	41.23	42.58	44.55	48.15
13.50	20.57	25.41	27.85	29.44	32.67	35.85	38.92	41.83	43.20	45.19	48.83
14.00	21.45	26.13	28.50	30.07	33.26	36.42	39.48	42.40	43.78	45.79	49.48
14.50	22.29	26.81	29.14	30.68	33.82	36.96	40.02	42.95	44.33	46.35	50.09
15.00	23.09	27.47	29.75	31.26	34.36	37.48	40.53	43.47	44.85	46.89	50.67
*Girls ages [years]*											
7.00	7.89	13.72	16.71	18.56	22.01	25.04	27.69	30.01	31.04	32.48	34.99
7.50	8.71	14.84	17.85	19.70	23.15	26.20	28.89	31.25	32.31	33.81	36.41
8.00	9.73	16.04	19.02	20.85	24.26	27.31	30.03	32.44	33.52	35.06	37.74
8.50	10.93	17.28	20.20	22.00	25.37	28.41	31.15	33.60	34.71	36.28	39.05
9.00	12.24	18.52	21.36	23.12	26.46	29.50	32.27	34.76	35.90	37.52	40.38
9.50	13.59	19.69	22.46	24.19	27.49	30.54	33.34	35.88	37.04	38.70	41.66
10.00	14.87	20.74	23.43	25.12	28.39	31.45	34.27	36.86	38.05	39.75	42.79
10.50	15.99	21.62	24.25	25.91	29.15	32.20	35.06	37.69	38.90	40.64	43.77
11.00	16.95	22.35	24.92	26.55	29.76	32.82	35.71	38.37	39.61	41.39	44.60
11.50	17.73	22.93	25.44	27.05	30.24	33.31	36.22	38.93	40.18	42.00	45.29
12.00	18.32	23.36	25.82	27.41	30.59	33.67	36.60	39.35	40.63	42.49	45.86
12.50	18.75	23.64	26.06	27.64	30.81	33.90	36.87	39.66	40.96	42.86	46.32
13.00	19.04	23.81	26.20	27.77	30.93	34.04	37.04	39.88	41.21	43.15	46.70
13.50	19.22	23.89	26.26	27.82	30.98	34.11	37.15	40.03	41.39	43.38	47.02
14.00	19.35	23.93	26.27	27.82	30.99	34.14	37.22	40.16	41.54	43.57	47.32
14.50	19.45	23.95	26.28	27.82	31.00	34.18	37.30	40.29	41.71	43.79	47.64
15.00	19.55	23.98	26.30	27.84	31.03	34.24	37.41	40.47	41.92	44.06	48.03

**Table 4:** Reference percentiles (P01 refers to the 1st percentile; other percentiles are denoted similarly) for muscular endurance remotely assessed by the Push-up test (number of repetitions in 40s) for children and adolescents from the COMO-Study (Boys: N=549; Girls: N=584).

	P01	P05	P10	P15	P30	P50	P70	P85	P90	P95	P99
*Boys ages [years]*											
7.00	3.20	4.33	5.05	5.59	6.85	8.35	10.09	12.04	13.07	14.72	18.24
7.50	3.30	4.49	5.24	5.80	7.09	8.60	10.34	12.25	13.25	14.84	18.17
8.00	3.39	4.65	5.43	6.00	7.32	8.85	10.57	12.45	13.43	14.96	18.13
8.50	3.48	4.80	5.61	6.20	7.54	9.08	10.80	12.65	13.60	15.08	18.11
9.00	3.57	4.95	5.78	6.39	7.76	9.31	11.02	12.84	13.76	15.20	18.11
9.50	3.66	5.10	5.96	6.57	7.96	9.53	11.23	13.02	13.93	15.32	18.13
10.00	3.75	5.24	6.12	6.76	8.17	9.73	11.43	13.20	14.08	15.45	18.15
10.50	3.83	5.38	6.29	6.93	8.36	9.94	11.62	13.37	14.24	15.57	18.19
11.00	3.92	5.52	6.45	7.11	8.55	10.13	11.81	13.53	14.39	15.69	18.23
11.50	4.00	5.65	6.61	7.28	8.74	10.33	11.99	13.69	14.53	15.81	18.28
12.00	4.08	5.79	6.76	7.44	8.92	10.51	12.17	13.85	14.67	15.92	18.34
12.50	4.16	5.92	6.92	7.61	9.10	10.69	12.34	14.00	14.81	16.04	18.39
13.00	4.24	6.05	7.07	7.77	9.27	10.87	12.51	14.15	14.95	16.15	18.46
13.50	4.32	6.18	7.22	7.93	9.44	11.04	12.67	14.29	15.08	16.27	18.52
14.00	4.40	6.31	7.36	8.08	9.61	11.20	12.83	14.44	15.21	16.38	18.58
14.50	4.48	6.44	7.51	8.23	9.77	11.37	12.98	14.57	15.34	16.49	18.65
15.00	4.56	6.57	7.65	8.39	9.93	11.53	13.13	14.71	15.47	16.59	18.72
*Girls ages [years]*											
7.00	3.24	4.96	5.95	6.64	8.17	9.81	11.54	13.29	14.16	15.47	18.01
7.50	3.26	4.95	5.93	6.62	8.13	9.76	11.47	13.22	14.08	15.38	17.90
8.00	3.27	4.95	5.92	6.61	8.10	9.72	11.42	13.15	14.00	15.29	17.80
8.50	3.28	4.95	5.91	6.59	8.07	9.67	11.36	13.08	13.92	15.20	17.70
9.00	3.30	4.94	5.90	6.57	8.04	9.63	11.30	13.01	13.85	15.12	17.60
9.50	3.31	4.94	5.89	6.55	8.01	9.59	11.25	12.94	13.78	15.04	17.50
10.00	3.32	4.94	5.88	6.54	7.98	9.55	11.19	12.88	13.71	14.96	17.41
10.50	3.33	4.93	5.87	6.52	7.95	9.51	11.14	12.81	13.64	14.89	17.32
11.00	3.34	4.93	5.85	6.50	7.93	9.47	11.09	12.75	13.57	14.81	17.23
11.50	3.35	4.93	5.84	6.49	7.90	9.43	11.04	12.69	13.51	14.74	17.14
12.00	3.36	4.92	5.83	6.47	7.87	9.39	10.99	12.63	13.44	14.67	17.06
12.50	3.37	4.92	5.82	6.46	7.85	9.36	10.95	12.58	13.38	14.60	16.98
13.00	3.37	4.92	5.81	6.44	7.82	9.32	10.90	12.52	13.32	14.53	16.90
13.50	3.38	4.91	5.80	6.43	7.80	9.29	10.86	12.46	13.26	14.47	16.82
14.00	3.39	4.91	5.79	6.41	7.77	9.25	10.81	12.41	13.20	14.40	16.74
14.50	3.39	4.90	5.78	6.39	7.75	9.22	10.77	12.36	13.14	14.34	16.67
15.00	3.40	4.90	5.77	6.38	7.72	9.18	10.73	12.31	13.09	14.28	16.59

**Table 5:** Reference percentiles (P01 refers to the 1st percentile; other percentiles are denoted similarly) for muscular endurance remotely assessed by the Sit-up test (number of repetitions in 40s) for children and adolescents from the COMO-Study (Boys: N=547; Girls: N=586).

	P01	P05	P10	P15	P30	P50	P70	P85	P90	P95	P99
*Boys ages [years]*											
7.00	1.51	4.32	6.29	7.69	10.64	13.60	16.46	19.17	20.44	22.29	25.66
7.50	1.73	4.77	6.81	8.23	11.20	14.18	17.07	19.81	21.10	22.97	26.42
8.00	2.00	5.27	7.34	8.78	11.75	14.73	17.63	20.40	21.70	23.60	27.09
8.50	2.35	5.80	7.90	9.33	12.29	15.26	18.16	20.93	22.24	24.15	27.68
9.00	2.77	6.37	8.47	9.89	12.82	15.77	18.66	21.42	22.73	24.65	28.21
9.50	3.27	6.96	9.04	10.44	13.34	16.26	19.12	21.88	23.19	25.12	28.68
10.00	3.83	7.56	9.61	10.99	13.84	16.72	19.56	22.30	23.61	25.53	29.10
10.50	4.45	8.16	10.16	11.51	14.31	17.15	19.96	22.68	23.98	25.89	29.46
11.00	5.09	8.73	10.69	12.01	14.74	17.53	20.31	23.00	24.29	26.19	29.74
11.50	5.73	9.28	11.18	12.46	15.14	17.88	20.61	23.27	24.54	26.42	29.95
12.00	6.35	9.79	11.64	12.89	15.50	18.18	20.86	23.48	24.74	26.60	30.09
12.50	6.94	10.28	12.07	13.28	15.83	18.45	21.08	23.66	24.90	26.73	30.18
13.00	7.51	10.73	12.47	13.66	16.14	18.70	21.28	23.81	25.03	26.84	30.25
13.50	8.04	11.17	12.86	14.01	16.43	18.94	21.46	23.95	25.15	26.93	30.29
14.00	8.55	11.58	13.22	14.34	16.70	19.15	21.63	24.07	25.25	27.01	30.32
14.50	9.03	11.97	13.57	14.66	16.96	19.36	21.79	24.18	25.34	27.07	30.33
15.00	9.48	12.34	13.89	14.95	17.20	19.55	21.92	24.28	25.41	27.11	30.32
*Girls ages [years]*											
7.00	3.18	6.02	7.64	8.76	11.15	13.65	16.21	18.74	19.97	21.80	25.28
7.50	3.43	6.34	7.99	9.12	11.53	14.04	16.60	19.13	20.36	22.19	25.65
8.00	3.69	6.66	8.33	9.47	11.89	14.42	16.98	19.51	20.73	22.55	26.00
8.50	3.95	6.97	8.66	9.81	12.24	14.77	17.34	19.86	21.08	22.89	26.32
9.00	4.20	7.28	8.97	10.13	12.58	15.11	17.67	20.19	21.40	23.20	26.61
9.50	4.43	7.56	9.27	10.44	12.89	15.43	17.98	20.49	21.69	23.48	26.85
10.00	4.65	7.83	9.55	10.73	13.18	15.72	18.26	20.74	21.94	23.71	27.04
10.50	4.85	8.07	9.81	10.98	13.45	15.97	18.49	20.95	22.13	23.88	27.16
11.00	5.02	8.28	10.04	11.22	13.67	16.18	18.68	21.12	22.28	24.00	27.22
11.50	5.16	8.47	10.24	11.42	13.87	16.36	18.84	21.24	22.38	24.07	27.22
12.00	5.29	8.64	10.41	11.59	14.04	16.51	18.95	21.31	22.43	24.09	27.17
12.50	5.38	8.78	10.56	11.75	14.18	16.63	19.03	21.35	22.45	24.07	27.06
13.00	5.46	8.90	10.69	11.88	14.30	16.72	19.09	21.36	22.43	24.01	26.92
13.50	5.52	9.01	10.80	11.99	14.40	16.79	19.12	21.34	22.39	23.92	26.75
14.00	5.56	9.10	10.91	12.09	14.48	16.85	19.14	21.31	22.33	23.83	26.57
14.50	5.60	9.19	11.00	12.18	14.56	16.90	19.14	21.27	22.27	23.72	26.38
15.00	5.64	9.27	11.09	12.27	14.63	16.94	19.15	21.23	22.20	23.61	26.20

## Discussion

This study provides the first reference values/percentiles for remotely assessed physical fitness tests of German children and adolescents aged 7 to 15 years. By applying rigorous statistical modeling (GAMLSS)^24^ to data collected through the DigiMot test profile, our findings offer age- and sex-specific reference percentiles for the key fitness components of coordination and muscular endurance^13^ under home-based remote assessment conditions. These results represent a novel contribution to the field of remote physical fitness monitoring, particularly in light of the need in scalable, digital health tools for children and adolescents^32^. The observed developmental trajectories align mostly with established percentiles^18–20^ and literature^33,34^, wherein physical fitness generally improves with age to varying degrees. Boys’ performance in strength-based tasks, such as Sit-ups and Push-ups, continues to improve through late adolescence, while girls’ performance typically plateaus around the age of 11 years^33,34^. This trend is also reflected in our percentiles, with the exception of the girls’ Push-up percentiles, which show a steady decline or stagnation with age. In contrast to previous findings from field-based assessments, where girls’ upper-body strength typically increases until around age 11 or 12 before plateauing^20,33^, our data reveal a continuous decline starting already at age 7. This pattern is unlikely to be explained by biological maturation, which would rather predict initial improvement due to growth and neuromuscular development. We therefore cautiously hypothesize that methodological and motivational factors may contribute in remote testing contexts. Girls may have been less familiar or confident performing strength-related tasks without in-person supervision, and differences in home environments (e.g., available space or camera angle) could have influenced the execution and scoring of Push-ups. Furthermore, reduced social facilitation in remote settings may lead to lower effort or early task termination compared with controlled field conditions. These factors warrant further investigation in future studies. In coordination tasks, such as Jumping Sideways, both boys and girls demonstrate significant performance gains. However, boys continue to improve through late adolescence, whereas girls’ performance tends to plateau around age 12^34^. Our percentiles for the Jumping Sideways align with these established developmental trajectories.

A direct comparison of the DigiMot reference percentiles with the established MoMo reference percentiles^20^ reveals several notable differences. It should be emphasized that both sets of reference values are method- and context-specific and therefore not directly interchangeable or substitutable. For Push-ups and Sit-ups, the gap between the percentiles increases with age, with performance values in the MoMo percentiles consistently higher than those in the DigiMot percentiles. On average across all percentiles and age groups, the DigiMot percentiles for Push-ups are 2.2 repetitions lower for boys and 2.3 repetitions lower for girls. For Sit-ups, the DigiMot percentiles are, on average, 3.0 repetitions lower for boys and 2.6 repetitions lower for girls. For the Jumping sideways, the DigiMot percentiles are higher up to age 12 (particularly at age 7) after which the MoMo percentiles become increasingly higher. On average, boys performed 1.4 and girls 0.5 more repetitions in the DigiMot percentiles. These differences are likely attributable to variations in standardization, testing conditions, and supervision between the MoMo and DigiMot test protocols. In particular, methodological characteristics inherent to remote testing may have amplified these effects. Factors such as interrater reliability, camera positioning, and the visibility of movement execution can influence scoring accuracy and comparability. Moreover, the home environment introduces additional variability in space, lighting, and participant focus, potentially affecting performance outcomes. Participants may also find it more difficult to reach maximal effort without the social facilitation and structured atmosphere typically present in supervised, laboratory-based settings. Although the option to record and review assessments enhances objectivity, these contextual and methodological influences may still cause systematic deviations from standardized field-based assessments. Another possible explanation for the observed differences is the inclusion of a higher proportion of lower-performing children and adolescents in the DigiMot study, potentially due to increased accessibility and willingness to participate in remote testing. Overall, these methodological differences must be considered when comparing performance outcomes and preclude direct substitution between the DigiMot and MoMo test profiles.

Nonetheless, the DigiMot test’s remote format introduces a valuable dimension to fitness testing by expanding access beyond traditional, field-based settings. As previous research has shown, remote assessments can increase inclusivity by reducing barriers related to geography, physical limitations, or scheduling constraints which are factors that often exclude specific population groups from conventional testing environments^32,35^. Moreover, the ability to video-record and subsequently re-evaluate the fitness assessments both qualitatively and quantitatively can enhance the quality of the collected data. This makes the DigiMot test particularly suitable for use in both clinical and educational contexts as a flexible and accessible monitoring solution. However, the DigiMot test’s remote format also presents some drawbacks that must be considered. Due to the videoconferencing setup, the participant’s camera must be adjusted for each test; otherwise, the full test procedure may not be visible to the test administrators, potentially leading to data collection errors. In addition, errors are more difficult to correct due to the limited visual perspective, and motivating participants is more challenging compared to in-person testing.

Beyond its practical utility, this study makes an important methodological contribution by broadening the tools available for assessing youth fitness. Remote fitness testing can provide greater flexibility and scalability for researchers and practitioners, especially in population-based surveillance or longitudinal studies. By establishing reference percentiles for remotely administered fitness tests, our results serve as a valuable benchmark for future studies and for public health efforts aimed at promoting fitness in youth. While most prior research on remote fitness testing has focused on older adults, this study helps fill a significant gap by providing data for children and adolescents^32^. Moreover, the remote setting holds potential for reaching underrepresented groups who may face barriers to in-person assessments, such as individuals with impaired mobility, health-constraints, or those who are more comfortable in a familiar home environment^35^. As remote health solutions continue to evolve, further research should aim to extend this approach to a broader array of fitness components, particularly cardiovascular endurance, which is rarely assessed in remote settings^32^ and is an important marker for health^36^. Given the spatial and logistical constraints associated with conducting such tests via videoconferencing, methodological innovations will be required to ensure reliability, feasibility, and validity. Continued efforts in this direction can support the development of comprehensive, remote-compatible fitness test profiles for both younger and older populations.

By providing normative reference data for remote physical fitness testing, this study contributes to the advancement of scalable health monitoring systems in children and adolescents. The DigiMot test could be integrated into school-based physical education programs or community health initiatives to regularly track fitness levels and identify early declines in physical development. Such remote, low-threshold approaches offer an opportunity to complement national surveillance systems and to support evidence-based interventions promoting physical activity and fitness. In the long term, the availability of standardized remote assessments may help inform educational and health policy strategies aimed at reducing inequalities in access to fitness evaluation and promoting active lifestyles from early childhood onward.

**Fig. 4:**
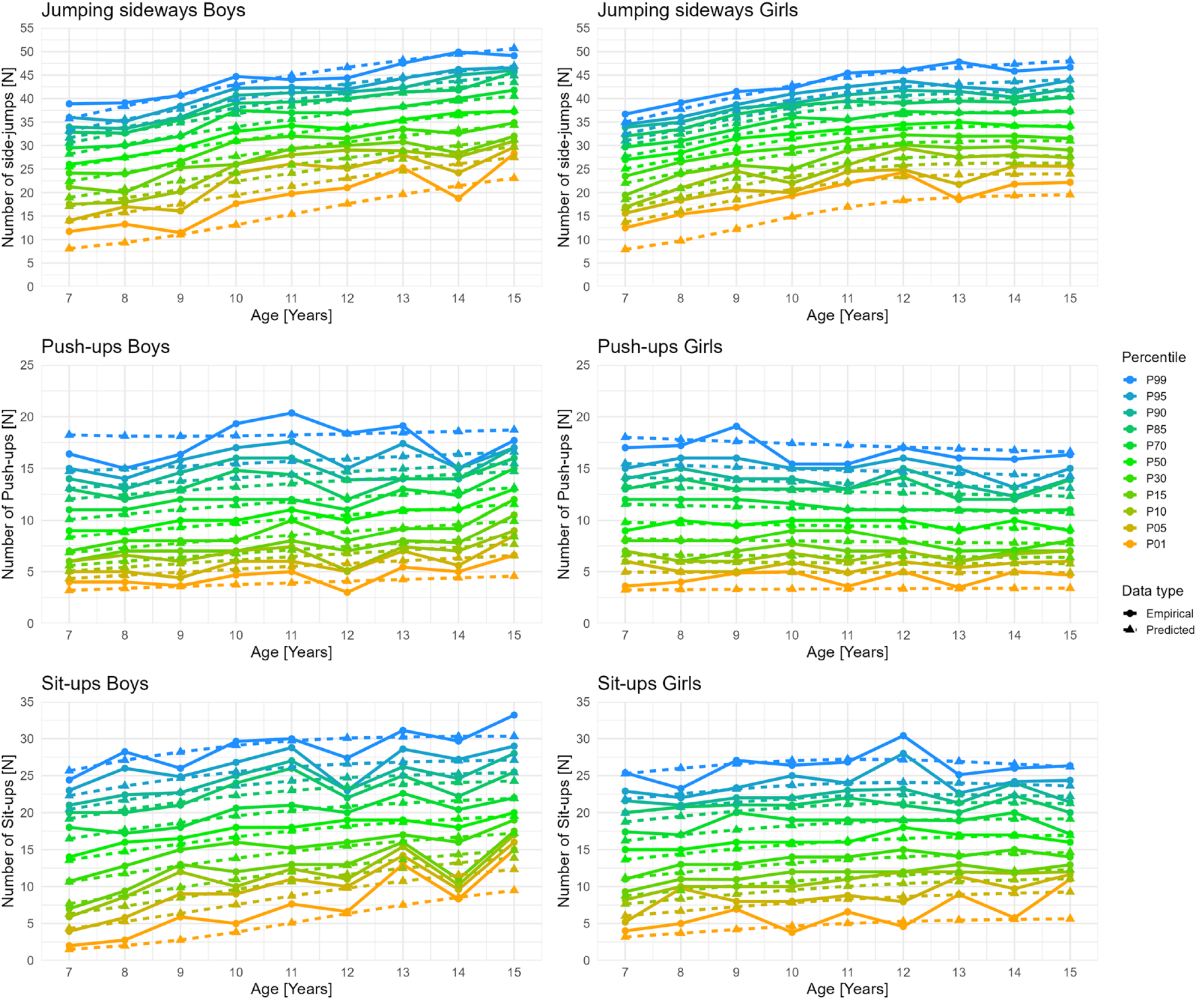
Comparison between empirical and predicted percentiles for Jumping sideways, Push-ups, and Sit-ups in boys and girls aged 7 to 15 years.

Although we were able to include 1149 participants in our study, which is, to our knowledge, the largest to date employing remote physical fitness testing, several limitations should be noted. First, the distribution of participants across age groups was uneven, with a noticeable decline in sample size beginning at age 13 and continuing through late adolescence (Table 2). Therefore, we decided to include all participants (7 to 17 years) in the analysis but report reference percentiles only for ages 7 to 15 years. Nonetheless, this imbalance reduces the reliability of the reference values for older adolescents, as reflected in the increasing discrepancies between predicted and empirical percentiles in this age range (Figure 4). Furthermore, we observed greater differences between predicted and empirical values at the extreme percentiles (1–10 and 90–99), indicating reduced reliability in these ranges. These limitations highlight the need for cautious interpretation of the percentile data, particularly for less-represented age groups and at the distribution tails. Accordingly, values at the extreme percentiles should be interpreted primarily at the group level, as estimates for individual assessment may be less stable, particularly in less-represented age groups. Although we applied weighting in the modeling process to enhance the representativeness of the percentiles, it is important to note that the original weights were not explicitly constructed for the selected subsample and may not fully correct for potential selection biases. In particular, our subsample showed an overrepresentation of children aged 8 to 12 years and an underrepresentation of older adolescents (ages 14–17) compared to the age distribution in the fully weighted COMO study sample. Because no post-stratification or recalibrated weights were available for the substudy sample, this imbalance could not be corrected through weighting. As such, results should be interpreted with caution, particularly for age-specific inferences, and may not be fully generalizable to the entire 7- to 15-year-old population.

## Conclusion

The findings confirm the feasibility and scalability of remote fitness testing in youth, supporting its role as a complementary approach to traditional field-based assessments. By providing standardized percentile benchmarks, DigiMot can facilitate longitudinal monitoring, evaluation of school-based interventions, and the early identification of children with below-average fitness levels in both educational and health contexts. These results highlight the potential of digital tools like DigiMot to broaden access to fitness assessment while maintaining methodological rigor and data quality.

Beyond its methodological contribution, DigiMot offers concrete opportunities for application in schools and community health promotion. Its scalable and accessible format allows for large-scale monitoring and can inform targeted, evidence-based interventions. For instance, children identified with lower fitness levels could benefit from additional or adapted physical education sessions conducted in smaller, supportive groups. While the DigiMot test cannot distinguish between developmental and lifestyle-related motor challenges, it provides a valuable screening tool to guide early and inclusive support measures. Furthermore, communicating assessment results to parents, along with practical recommendations and an emphasis on active parental role modeling, may help raise awareness of the importance of motor development and promote family-based physical activity. Collaboration with sports clubs and community programs could further strengthen these initiatives by offering structured opportunities for participation and reinforcement of motor skills.

Despite certain limitations in sample representativeness and percentile reliability among older adolescents, the reference values established here provide a robust foundation for future research and practical application. As digital health technologies continue to evolve, DigiMot can serve as a model for integrating remote fitness assessment into public health surveillance and preventive strategies. Future studies should aim to validate remote testing protocols in more diverse populations, examine their longitudinal stability, and expand the approach to additional fitness domains such as cardiovascular endurance. Through these efforts, remote fitness testing may become an integral component of inclusive, data-driven strategies to promote physical activity and motor competence among children and adolescents.

## Data Availability

The datasets generated during and/or analyzed during the current study are available from the corresponding author on reasonable request.
